# Molecular transmission network analysis of newly diagnosed HIV-1 infections in Ningbo from 2018–2022

**DOI:** 10.3389/fmicb.2025.1701408

**Published:** 2025-11-19

**Authors:** Yue-Qi Yin, Yu-Hui Liu, Jing Zhu, Peng Shen, Yun-Peng Chen, Zhi-Qin Jiang, Hong-Bo Lin, Hong-Xia Ni, Ye-Xiang Sun

**Affiliations:** 1 Department of Data Center, Yinzhou District Center for Disease Control and Prevention, Ningbo, Zhejiang, China; 2 Ningbo Center for Disease Control and Prevention, Ningbo, Zhejiang, China; 3 Nanjing Jiangning Hospital, Nanjing, Jiangsu, China

**Keywords:** HIV-1, molecular transmission networks, transmission clusters, subtype, prevention strategies

## Abstract

**Introduction:**

Understanding molecular transmission patterns is critical for HIV prevention designed with key populations. This study aimed to characterize the molecular epidemiology, transmission networks, and underlying factors associated with HIV-1 transmission in Ningbo during 2018–2022.

**Methods:**

We analyzed data from 1,409 newly diagnosed people living with HIV who had successful genotyping. A maximum likelihood phylogenetic tree was constructed, and transmission clusters were identified using 1.3% distance and 0.9 bootstrap values. Multivariate logistic regression was applied to identify factors associated with clustered, large clusters (≥10 nodes) and fast-growing clusters.

**Results:**

Molecular analysis revealed 11 distinct HIV-1 subtypes and some unique recombinant forms (URFs), with CRF07_BC (41.6%) and CRF01_AE (33.2%) as the most prevalent. CRF07_BC consistently tended to form larger, more densely connected clusters, whereas CRF01_AE networks primarily exhibited sparse, fragmented distributions. Molecular transmission network analysis identified 9 large clusters and 12 fast-growing clusters. HIV-1 subtypes were associated with the large clusters and fast-growing clusters. CRF07_BC formed larger clusters (aOR = 7.80, 95%CI: 4.70–13.49) and fast-growing clusters (aOR = 6.02, 95%CI: 3.80–9.78) compared to CRF01_AE. Temporally, the molecular transmission networks (MTNs) expanded rapidly in 2020–2021.

**Conclusion:**

This study elucidates the MTNs of HIV-1 in Ningbo, highlighting the role of subtype diversity and demographic traits in shaping transmission networks. Continuous monitoring of HIV-1 molecular subtypes among key populations may serve as feasible and focused prevention strategies to curb HIV transmission.

## 1 Introduction

Despite significant global efforts to control the transmission of HIV-1, Acquired Immune Deficiency Syndrome (AIDS) remains a major global public health issue. At the end of 2024, an estimated 40.8 million (37.0–45.6 million) people were living with HIV globally, including 1.4 million (1.1–1.8 million) children (0–14 years old) and 39.4 million (35.7–44.0 million) adults (15 + years old) (UNAIDS, Global HIV, 2025). In China, as of Dec. 31, 2024, there were 1,355,017 reported cases of people living with HIV, with 491,437 reported deaths (National Center for STD/AIDS Control, Chinese Center for Disease Control and Prevention, 2025). Over the past decade, the incidence of AIDS has shown a sustained upward trend across most regions of China, characterized by continuous spread from south to north and west to east, coupled with increasing disease intensity and a growing burden of illness (Wang et al., 2019). From 1990 to 2021, the incidence, mortality, and disability-adjusted life years (DALYs) of HIV increased in the general population, with age-standardized incidence, mortality, and DALY rates rising at average annual rates of 0.051, 0.056, and 2.629, respectively (He et al., 2025). These trends underscore the critical need for enhanced HIV prevention and control strategies to curb transmission, reduce incidence, and alleviate the escalating disease burden on affected populations.

On June 8, 2021, The Joint United Nations Program on HIV/AIDS (UNAIDS) put forward the “95–95-95 targets” (95% of all PLWH knowing their status, 95% of those diagnosed accessing treatment, and 95% of those on treatment achieving viral suppression) to be achieved by 2025 (United Nations General Assembly, 2021). The “95–95-95” targets are not only the global action plan for the response to HIV but also the core indicators for measuring progress. Their significance lies in transforming AIDS from an “incurable epidemic” into a “controllable chronic disease” through systematic and multi-level prevention and control measures, and ultimately achieving the vision of ending the AIDS epidemic. Following this, WHO has rolled out a series of action plans and guidelines to advance these goals (World Health Organization, 2022). Globally, as of 2024, 87% (69–98%) of all PLWH knew their HIV status. Among those aware of their status, 89% (71–98%) were accessing treatment, and 94% (75–98%) of those on treatment achieved viral suppression (UNAIDS, Global HIV, 2025). In China, since implementing the “treat-all” strategy (initiating antiretroviral therapy (ART) regardless of CD4 cell count) in 2016, ART coverage has further improved (Bai et al., 2020). By the end of 2022, China’s progress toward the “95-95-95 targets” stood at 84-93-97% (Ye et al., 2024). While significant progress has been made in scaling up treatment coverage and improving treatment outcomes, a substantial gap persists in case detection compared to the global targets. Addressing this gap remains a critical challenge.

Molecular transmission networks (MTNs), constructed from HIV genetic sequence data, have emerged as critical tools for dissecting HIV-1 transmission dynamics. Initially formalized as a prevention strategy by the U. S. CDC in 2018 (Oster et al., 2018), MTNs were firstly adapted into China’s guidelines for monitoring and intervention by the National Center for AIDS/STD Control and Prevention in 2019 (China CDC, 2019). By reconstructing evolutionary and transmission-related associations among viral sequences, MTNs facilitate the identification of HIV transmission clusters and the delineation of ancestral-contemporary infection relationships. Their applications have expanded beyond fundamental research, now encompassing early case detection of HIV infections, long-term surveillance of HIV drug resistance, and the design of precision-focused intervention strategies (Zhao et al., 2022; Han et al., 2020; Liu et al., 2020). Currently, MTNs play a pivotal role in the operational control of HIV epidemics: they assist in determining the timing and geographical locations of new HIV infections, quantifying HIV transmission velocity, and evaluating the efficacy of HIV prevention interventions. By integrating genetic sequence analysis with real-world patterns of HIV spread, MTNs provide evidence-based and actionable insights to inform strategies aimed at curbing the transmission of HIV.

Zhejiang Province, home to a population exceeding 60 million, reported 4,279 newly diagnosed HIV infections in 2022 alone (He et al., 2025). Epidemiological data from 2018 indicate an HIV incidence rate of 1.67 per 10,000 population and an HIV testing positivity rate of 45.1 per 10,000 population in the province (Chen et al., 2021). These figures underscore the persistent challenges in controlling HIV transmission in Zhejiang, particularly given its status as a major economic hub with substantial population mobility. Zhejiang Province has conducted a series of studies leveraging MTNs to investigate multiple dimensions of HIV transmission, including network characteristics across distinct populations and regions, and the evaluation of HIV intervention efficacy. These research efforts have yielded evidence-based contributions to the regional control of HIV transmission (Chen et al., 2021; Dai et al., 2024; Chen et al., 2024). Ningbo, a coastal city in eastern Zhejiang Province, possesses the longest coastline within the province and is home to the Ningbo-Zhoushan Port—one of the world’s largest cargo throughput ports, which has maintained this leading position for 16 consecutive years. With a permanent population of approximately 10 million (accounting for one-sixth of Zhejiang’s total population), Ningbo also grapples with a distinct HIV epidemic: the city recorded an HIV incidence rate of 1.24 per 10,000 population in 2018 (Chen et al., 2021). In our previous work, we performed an analysis of the HIV molecular transmission network among men who have sex with men (MSM) in Ningbo and further explored drug resistance-associated HIV transmission patterns through complementary network-based studies (Hong et al., 2018; Hong et al., 2023; Shi et al., 2024). Building on these foundational investigations, the present study aims to comprehensively characterize the HIV molecular transmission network in Ningbo spanning the period 2018–2022. A specific focus will be placed on analyzing the influencing factors underlying the formation of large-scale transmission networks and the expansion of rapidly growing networks in this region. Findings from this work are expected to provide actionable insights for designing focused HIV intervention strategies.

## 2 Method

### 2.1 Study population and laboratory tests

This study enrolled all individuals aged ≥18 years who were newly diagnosed with HIV-1 infection in Ningbo from January 2018 to December 2022 and had not initiated antiretroviral therapy (ART). Written informed consent was obtained from all participants prior to study inclusion. Blood samples were processed following standardized protocols, and the partial *pol* gene segment (HXB2: 2253–3,283) was sequenced using a previously validated protocol (Hong et al., 2023). Only sequences exceeding 1,000 base pairs in length were included for subsequent phylogenetic and molecular transmission network analyses.

### 2.2 Sequences analysis

Raw sequence reads were assembled and aligned using ChromasPro 1.6 (Technelysium Pty Ltd.), and the Gene Cutter online tool.^1^ Reads with <1,000 nucleotides in length or >1.5% ambiguous bases were excluded. Duplicate sequences were identified and removed using the online tool ElimDupes.^2^ To facilitate subtype classification and phylogenetic analysis, all remaining sequences were submitted to the LANL HIV Database (see text footnote 1, respectively). For each query sequence, the top 10 genetically similar reference sequences were downloaded from the database. These reference sequences, along with the query sequences, were then used to construct maximum likelihood (ML) phylogenetic trees using FastTree v2.1.10 under the GTR + G + I nucleotide substitution model. Local support values for clades were calculated via the Shimodaira–Hasegawa approximate likelihood ratio test (SH-aLRT) to assess phylogenetic robustness. The reference sequences cited in this study are provided in Supplementary Table S1.

Drug-resistance mutations were identified and scored using the Stanford HIVdb Program,^3^ following the 2009 WHO Surveillance Drug Resistance Mutations (SDRMs) list (Bennett et al., 2009). The database automatically assigns a resistance score to each detected mutation based on its impact on drug susceptibility, with scores stratified as: 0–9 (sensitive), 10–14 (potential low-level resistance), 15–29 (low-level resistance), 30–59 (moderate resistance), and ≥60 (high-level resistance). For each sample, the highest score across all detected mutations was selected as its representative resistance level. Samples with a total score >15 were classified as resistant, whereas those with scores ≤15 were deemed sensitive.

### 2.3 HIV-1 transmission network analysis

Potential transmission clusters were extracted from the phylogenetic tree using Cluster Picker v1.2.5, with inclusion criteria of bootstrap support values ≥90% and maximum pairwise genetic distance ≤1.3% nucleotide substitutions per site (Chen et al., 2024; Hong et al., 2023), detailed information is provided in Supplementary Figure S1. For all extracted sequences, Tamura-Nei 93 pairwise genetic distances were calculated using HyPhy 2.2.4, and clusters were required to contain at least two sequences. The transmission network was visualized using Cytoscape v3.7.0, where edges represent potential transmission relationships between connected subjects, and network degree (the number of links per sequence node) was used to quantify transmission propensity—higher degree values indicating a greater likelihood of viral transmission. To guide HIV intervention strategies, we further characterized cluster size and growth dynamics: large clusters were defined as those containing ≥10 sequences, small clusters were defined as those containing 2 to 9 sequences, while fast-growing clusters were defined as exhibiting a ≥ 5-sequence increase in node count within a 1-year sequence collection period (Yin et al., 2021; Wertheim et al., 2018; Shi et al., 2024). The baseline transmission network was constructed using samples collected during the first 2 years of the study.

### 2.4 Statistical analysis

Statistical analyses were conducted using R version 4.4.2. Continuous variables with non-normal distributions were summarized using descriptive statistics, including interquartile ranges (IQR) and medians, while categorical variables were described with frequencies and percentages. To identify factors associated with transmission within molecular clusters, large molecular clusters, and fast-growing clusters, univariate and multivariate logistic regression models were employed. Variables showing significance in univariate analysis (*p* < 0.2) were included in the multivariate model. All statistical tests were two-tailed, with a significance level set at *p* < 0.05.

## 3 Result

### 3.1 Study population

Between January 2018 and December 2022, we enrolled 1,518 individuals newly diagnosed with HIV-1 infection. Of these, 1,409 (92.8%) underwent successful HIV-1 genotyping and were included in subsequent MTNs analyses. Demographic characteristics revealed male predominance (85.0%), a median age of 40.6 years (interquartile range [IQR]: 28.2–53.5 years; mean ± SD: 41.9 ± 15.2 years), and 95.7% self-identifying as Han Chinese. Marital status distribution showed 42.7% married and 37.4% single. The highest age proportion was in the 25–35 group (25.9%), followed by individuals aged >55 years (22.4%). Educational attainment was low, with only 23.0% holding a college degree or higher. Occupational distribution indicated service-sector employment as the primary occupation (43.8%). Transmission routes included heterosexual contact (53.7%), exceeding homosexual contact (45.9%). Discovery pathways were dominated by general outpatient clinics (22.0%), preoperative examinations (20.9%), and counseling/testing services (20.3%). At diagnosis, 37.8% of individuals had CD4 counts <200 cells/μL. Geographic distribution highlighted local residency (72.3%), with Ningbo’s Yinzhou (20.6%) and Haishu (19.0%) districts accounting for the highest proportions. Subtype distribution exhibited significant regional variation (*p* < 0.001; Supplementary Table S2).

### 3.2 Molecular epidemiology and drug resistance characteristics

We employed a 1,030-bp fragment of the partial *pol* gene to construct a ML phylogenetic tree (Supplementary Figure S2). A total of 12 distinct HIV-1 genotypes were identified, with CRF07_BC (41.6%) and CRF01_AE (33.2%) being the predominant subtypes. The remaining genotypes included CRF08_BC (6.7%), CRF55_01B (5.7%), URF (4.6%), CRF85_BC (2.4%), B (2.3%), CRF57_BC (1.5%), C (0.8%), CRF59_01B (0.4%), CRF67_01B (0.4%), and CRF68_01B (0.2%).

According to the 2009 WHO SDRMs list, 7.9% (*n* = 111/1,409) of sequences exhibited SDRMs, including 14 NRTI-associated mutations, 15 NNRTI-associated mutations, and 2 PI-associated mutations (Supplementary Figure S3A). Notably, 17 antiretroviral drugs showed varying levels of resistance, with high-grade resistance detected against EFV (efavirenz), NVP (nevirapine), FTC (emtricitabine), and 3TC (lamivudine) (Supplementary Figure S3B). Subtype-specific resistance patterns were also observed (*p =* 0.015; Supplementary Table S2).

### 3.3 Characteristics of HIV-1 transmission networks

A total of 168 HIV-1 molecular transmission networks were identified, encompassing 692 individuals (49.1% of the 1,409 successfully genotyped individuals) and 2,063 edges. Network sizes ranged from 2 to 139 nodes, with the majority (59.5%) comprising dyadic clusters (two nodes). Notably, 9 clusters contained ≥10 sequences (Supplementary Figure S4A). Node connectivity exhibited marked heterogeneity: 36.6% of nodes (253/692) formed single linkages, 49.3% (341/692) engaged in 2–10 linkages, and 14.2% (98/692) demonstrated extensive connectivity (>20 linkages) (Supplementary Figure S4B). Under a 1.5% genetic distance threshold, 30.0% of edges (618/2,063) exhibited minimal genetic divergence (<0.005), suggesting recent or ongoing transmission events (Supplementary Figure S4C).

**Figure 1:**
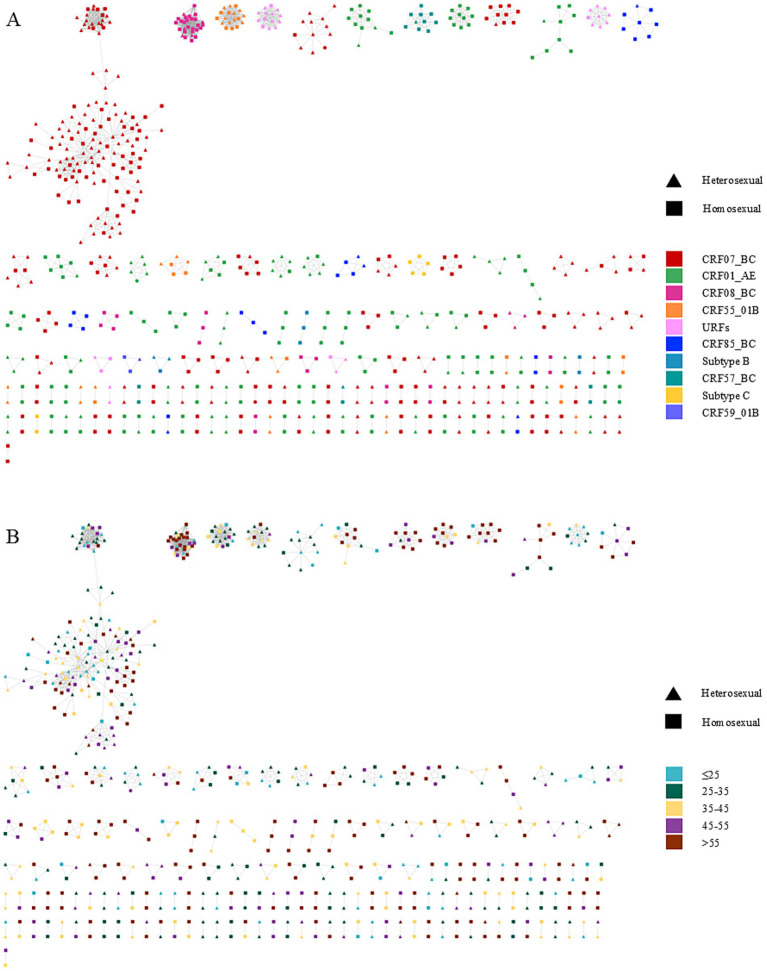
Molecular transmission network of people living with HIV in Ningbo, China (2018–2022). Clusters are ordered by size within each panel. Node shapes denote distinct transmission routes, and node colors represent participant characteristics, including HIV-1 genotypes and age groups. **(A)** corresponds to the subtype distribution of the network. **(B)** represents the age distribution of the network.

Figure 1A illustrates the MTNs stratified by different HIV-1 subtypes. CRF07_BC dominated cluster composition with 320 nodes forming 63 clusters, including 60 small clusters comprising 160 nodes. Similarly, CRF01_AE contributed 187 nodes across 62 clusters, with 60 small clusters containing 166 nodes. CRF08_BC formed 9 clusters (49 nodes), 8 of which were small (21 nodes). CRF55_01B was distributed across 12 clusters (45 nodes), including 11 small clusters (27 nodes). The URF subtype comprised 5 clusters (31 nodes), 4 of which were small (17 nodes). CRF57_BC formed 4 clusters (16 nodes), 3 of which were small (6 nodes). Remaining subtypes all formed small clusters: CRF85_BC (26 nodes/7 clusters), subtype B (8 nodes/3 clusters), subtype C (7 nodes/2 clusters), and CRF59_01B (3 nodes/1 cluster). The largest molecular cluster was CRF07_BC, which consisted of 126 males and 11 females, with the primary mode of transmission being homosexual transmission (60.9%), details showed in Table 1. Furthermore, the dominant drug resistance mutation in the largest MTNs was Q58E, whereas other resistance-associated mutations were either scattered across the network or confined to smaller clusters. The genotypic and age-specific drug resistance profiles of MTNs are characterized in Supplementary Figure S5. Analysis of individuals with potential transmission links.

**Table 1:** Characteristics of the large molecular transmission clusters.

Cluster number	Subtype	Genetic distances Mean [Median, IQR]	Age	Gender	Transmission route	Drug resistance	Mutation site	District
1	CRF07_BC	0.001 [0.008, 0.004–0.011]	A1(22), A2(37), A3(22), A4(28), A5(29)	M(127), *F*(11)	Hetero(54), Homo(84)	R(8), S(130)	Q58E(5) M184V(2) N83D(1)	Hiashu, Yinzhou
2	CRF08_BC	0.001 [0.007, 0.004–0.009]	A1(3), A2(1), A3(3), A4(4), A5(17)	M(21), *F*(7)	Hetero(23), Homo(5)	S(28)		Xiangshan
3	CRF55_01B	0.001 [0.005, 0.004–0.009]	A1(2), A2(10), A3(4), A4(2)	M(18)	Hetero(8), Homo(10)	R(1), S(17)	K65R(1) V179D(1)	Cixi
4	URFs	0.007 [0.007, 0.005–0.009]	A2(6), A3(5), A4(1), A5(2)	M(13), *F*(1)	Hetero(3), Homo(11)	S(14)		Beilun
5	CRF07_BC	0.009 [0.009, 0.007–0.011]	A1(5), A2(7)	M(12)	Hetero(1), Homo(10), Unknow(1)	S(12)		Cixi
6	CRF01_AE	0.008 [0.008, 0.005–0.010]	A1(2), A2(2), A3(3), A5(4)	M(10), F(1)	Hetero(8), Homo(2)	S(11)		Yinzhou
7	CRF57_BC	0.009 [0.010, 0.008–0.011]	A2(1), A4(3), A5(6)	M(4), *F*(6)	Hetero(9), Homo(1)	S(10)		Xiangshan, Haishu
8	CRF01_AE	0.002 [0.005, 0.004–0.010]	A3(2), A4(2), A5(6)	M(6), *F*(4)	Hetero(10)	R(1), S(9)	K103N(1)	Fenghua, Yinzhou
9	CRF07_BC	0.006 [0.009, 0.007–0.011]	A1(1), A3(1), A4(1), A5(7)	M(9), F(1)	Hetero(9), Homo(1)	R(2), S(8)	G73S(1) K103N(1)	Xiangshan

A1: <=25 years, A2: 25–35 years, A3: 35–45 years, A4: 45–55 years, A5: >55 years; M: male, F: female. Hetero: heterosexual contact, Homo: homosexual contact; R: resistance, S: sensitive.

Univariate and multivariate logistic regression models were used to compare sequences included in the transmission networks versus those not included. Results are presented in Figure 1; Supplementary Table S3. The multivariate analysis revealed that individuals aged >55 years were more likely to cluster compared with those aged ≤25 years [adjusted odds ratio (aOR) = 2.379, 95% confidence interval (CI) = 1.623–3.502, *p* < 0.001]. Compared with Han ethnic patients, those from other ethnic groups showed reduced odds of clustering (aOR = 0.494, 95% CI = 0.270–0.873, *p* = 0.018). Patients with CD4 counts >200 cells/μL exhibited higher odds of clustering than those with CD4 ≤ 200 cells/μL (aOR = 1.295, 95% CI = 1.023–1.641, *p* = 0.032). Temporally, cases diagnosed in 2020 (aOR = 1.571, 95% CI = 1.055–2.349, *p* = 0.027) and 2021 (aOR = 1.765, 95% CI = 1.208–2.589, *p* = 0.003) exhibited higher odds of being part of transmission clusters compared to 2018. In contrast, 2022 showed no statistically significant difference in clustering odds relative to 2018 (aOR = 1.242, 95% CI = 0.856–1.809, *p* = 0.256). Regarding viral genotypes, CRF07_BC (aOR = 1.806, 95% CI = 1.395–2.342, *p* < 0.001) and others (aOR = 1.577, 95% CI = 1.177–2.115, *p* = 0.002) showed stronger clustering tendencies than CRF01_AE.

### 3.4 Characterization of large clusters

Nine large clusters (≥10 nodes) were identified, comprising 251 individuals (220 males and 31 females). Among these clusters, homosexual transmission (50.2%) and heterosexual transmission (49.4%) accounted for nearly equal proportions, with other routes contributing minimally (0.4%). A total of 12 transmitted drug resistance (TDR) cases were distributed across four large clusters. Within the nine large clusters, three were predominantly located in Xiangshan district (Table 1). Residents from Xiangshan exhibited significantly higher odds of clustering compared with Haishu district (aOR = 2.219, 95% CI = 1.086–4.594, *p* = 0.030). Conversely, Ninghai (aOR = 0.242, 95% CI = 0.064–0.736, *p* = 0.020), Yuyao (aOR = 0.395, 95% CI = 0.195–0.778, *p* = 0.008), and Zhenhai (aOR = 0.307, 95% CI = 0.094–0.853, *p* = 0.033) showed reduced odds of clustering relative to Haishu. Regarding viral genotypes, individuals infected with CRF07_BC (aOR = 7.796, 95% CI = 4.699–13.486, *p* < 0.001) and others (aOR = 4.619, 95% CI = 2.613–8.417, *p* < 0.001) exhibited stronger clustering tendencies than those with CRF01_AE (Table 2). A summary of large cluster characteristics (Table 1) further highlighted that 3 out of 9 identified large clusters (encompassing 251 individuals) were dominated by CRF07_BC, including the largest cluster in this study. Additionally, URFs constituted a large transmission network comprising 14 sequences, with infected individuals primarily residing in Beilun District, Ningbo City—an area adjacent to Ningbo-Zhoushan Port, one of China’s largest seaports (Table 3).

**Table 2:** Factors influencing the inclusion of individuals into the large molecular transmission clusters.

Characteristic	Clustered *N* = 692 (%)	Included in a large cluster (≥10 node) *N* = 251 (%)	Univariate analysis	Multivariate analysis
			OR (95% CI)	OR (95% CI)
Gender				
Male	580 (83.82)	220 (87.65)	1.000 (Reference)	1.000 (Reference)
Female	112 (16.18)	31 (12.35)	0.626 (0.401–0.979)*	0.670 (0.382–1.153)
Age (group)				
<=25	89 (12.86)	35 (13.94)	1.000 (Reference)	
25–35	175 (25.29)	64 (25.50)	0.890 (0.526–1.504)	
35–45	119 (17.20)	40 (15.94)	0.781 (0.442–1.382)	
45–55	112 (16.18)	41 (16.33)	0.891 (0.502–1.581)	
>55	197 (28.47)	71 (28.29)	0.869 (0.519–1.455)	
Marital status				
Single	247 (35.69)	99 (39.44)	1.000 (Reference)	
Married	317 (45.81)	103 (41.04)	0.720 (0.509–1.017)	
Divorce or death	128 (18.50)	49 (19.52)	0.927 (0.598–1.437)	
Education level				
Primary school or below	203 (29.34)	81 (32.27)	1.000 (Reference)	
Junior high school	215 (31.07)	66 (26.29)	0.667 (0.446–0.990)*	
Senior high school or secondary vocational school	122 (17.63)	40 (15.94)	0.735 (0.459–1.177)	
Associate degree or above	152 (21.97)	64 (25.50)	1.095 (0.714–1.679)	
Ethnic group				
Han	671 (96.97)	245 (97.61)	1.000 (Reference)	
Others	21 (3.03)	6 (2.39)	0.696 (0.266–1.816)	
Affiliated Region				
Haishu	122 (17.63)	51 (20.32)	1.000 (Reference)	1.000 (Reference)
Jiangbei	22 (3.18)	7 (2.79)	0.650 (0.247–1.708)	0.409 (0.132–1.147)
Beilun	62 (8.96)	21 (8.37)	0.713 (0.377–1.349)	0.604 (0.302–1.189)
Zhenhai	30 (4.34)	6 (2.39)	0.348 (0.133–0.913)*	0.307 (0.094–0.853)*
Yinzhou	153 (22.11)	62 (24.70)	0.949 (0.585–1.538)	1.106 (0.637–1.925)
Fenghua	38 (5.49)	12 (4.78)	0.643 (0.297–1.392)	0.648 (0.266–1.515)
Xiangshan	57 (8.24)	31 (12.35)	1.660 (0.881–3.127)	2.219 (1.086–4.594)*
Ninghai	23 (3.32)	4 (1.59)	0.293 (0.094–0.913)*	0.242 (0.064–0.736)*
Yuyao	78 (11.27)	18 (7.17)	0.418 (0.221–0.790)**	0.395 (0.195–0.778)**
Cixi	89 (12.86)	31 (12.35)	0.744 (0.423–1.310)	0.690 (0.369–1.280)
Others	18 (2.60)	8 (3.19)	1.114 (0.411–3.018)	1.249 (0.414–3.725)
Region Type				
Local city	524 (75.72)	195 (77.69)	1.000 (Reference)	
Other cities in this province	24 (3.47)	10 (3.98)	1.205 (0.525–2.765)	
Other provinces	144 (20.81)	46 (18.33)	0.792 (0.535–1.173)	
Transmission route				
Heterosexual	302 (43.64)	124 (49.40)	1.000 (Reference)	1.000 (Reference)
Homosexual	387 (55.92)	126 (50.20)	0.693 (0.507–0.947)*	0.785 (0.530–1.160)
Others	3 (0.43)	1 (0.40)	0.718 (0.064–8.002)	-
Drug resistance				
Sensitive	644 (93.06)	239 (95.22)	1.000 (Reference)	1.000 (Reference)
Resistance	48 (6.94)	12 (4.78)	0.565(0.288–1.107)	0.487 (0.215–1.035)
HIV-1 genotypes				
CRF01_AE	187 (27.02)	21 (8.37)	1.000 (Reference)	1.000 (Reference)
CRF07_BC	320 (46.24)	160 (63.75)	7.905 (4.775–13.086)***	7.796 (4.699–13.486)***
Others	185 (26.73)	70 (27.89)	4.812 (2.797–8.278)***	4.619 (2.613–8.417)***
Sample year				
2018	70 (10.12)	21 (8.37)	1.000 (Reference)	
2019	51 (7.37)	18 (7.17)	1.273 (0.590–2.746)	
2020	144 (20.81)	52 (20.72)	1.319 (0.714–2.437)	
2021	215 (31.07)	82 (32.67)	1.439 (0.805–2.571)	
2022	212 (30.64)	78 (31.08)	1.358 (0.759–2.432)	
Occupation				
Service sector	310 (44.80)	118 (47.01)	1.000 (Reference)	
Worker	122 (17.63)	46 (18.33)	0.985 (0.639–1.517)	
Former	156 (22.54)	53 (21.12)	0.837 (0.560–1.253)	
Others	104 (15.03)	34 (13.55)	0.790 (0.494–1.264)	
STD Status				
Yes	149 (21.53)	51 (20.32)	1.000 (Reference)	
No	468 (67.63)	169 (67.33)	1.086 (0.737–1.600)	
Unknown	75 (10.84)	31 (12.35)	1.354 (0.765–2.396)	
Detection Method				
Counseling and testing	143 (20.66)	58 (23.11)	1.000 (Reference)	
Physical examination	65 (9.39)	26 (10.36)	0.977 (0.537–1.777)	
STD clinic	108 (15.61)	33 (13.15)	0.645 (0.380–1.094)	
Focused survey	76 (10.98)	30 (11.95)	0.956 (0.541–1.687)	
General outpatient clinic	151 (21.82)	60 (23.90)	0.966 (0.606–1.541)	
Preoperative examination	149 (21.53)	44 (17.53)	0.614 (0.378–0.990)*	
CD4 (cell/ul)				
<200	243 (35.13)	71 (29.34)	1.000 (Reference)	1.000 (Reference)
≥200	449 (64.87)	171 (70.66)	1.502 (1.071–2.107)*	1.324 (0.908–1.938)

*p < 0.05; **p < 0.01; ***p < 0.001.

**Table 3:** Factors influencing the inclusion of individuals into the fast-growing molecular transmission clusters.

Characteristic	Clustered N = 692^a^ (%)	Fast-growing networks ^a^*N* = 268 (%)	Univariate analysis	Multivariate analysis
			OR (95% CI)	OR (95% CI)
Gender				
Male	580 (83.82)	233 (86.94)	1.000 (Reference)	
Female	112 (16.18)	35 (13.06)	0.677 (0.439–1.043)	
Age (group)				
<=25	89 (12.86)	36 (13.43)	1.000 (Reference)	
25–35	175 (25.29)	67 (25)	0.913 (0.542–1.539)	
35–45	119 (17.20)	41 (15.30)	0.774 (0.439–1.365)	
45–55	112 (16.18)	46 (17.16)	1.026 (0.582–1.808)	
>55	197 (28.47)	78 (29.10)	0.965 (0.579–1.608)	
Marital status				
Single	247 (35.69)	103 (38.43)	1.000 (Reference)	1.000 (Reference)
Married	317 (45.81)	107 (39.93)	0.712 (0.505–1.005)	0.689 (0.471–1.008)
Divorce or death	128 (18.50)	58 (21.64)	1.158 (0.753–1.781)	1.454 (0.898–2.363)
Education level				
Primary school or below	203 (29.34)	88 (32.84)	1.000 (Reference)	
Junior high school	215 (31.07)	73 (27.24)	0.672 (0.452–0.990)*	
Senior high school or secondary vocational school	122 (17.63)	41 (15.30)	0.661 (0.415–1.055)	
Associate degree or above	152 (21.97)	66 (24.63)	1.003 (0.656–1.533)	
Ethnic group				
Han	671 (96.97)	262 (97.76)	1.000 (Reference)	
Others	21 (3.03)	6 (2.24)	0.624 (0.239–1.630)	
Affiliated Region				
Haishu	122 (17.63)	53 (19.78)	1.000 (Reference)	1.000 (Reference)
Jiangbei	22 (3.18)	8 (2.99)	0.744 (0.291–1.904)	0.570 (0.205–1.506)
Beilun	62 (8.96)	23 (8.58)	0.768 (0.410–1.438)	0.641 (0.328–1.238)
Zhenhai	30 (4.34)	8 (2.99)	0.473 (0.195–1.147)	0.463 (0.172–1.161)
Yinzhou	153 (22.11)	70 (26.12)	1.098 (0.680–1.772)	1.168 (0.696–1.966)
Fenghua	38 (5.49)	12 (4.48)	0.601 (0.278–1.300)	0.550 (0.234–1.243)
Xiangshan	57 (8.24)	31 (11.57)	1.552 (0.825–2.921)	1.659 (0.837–3.314)
Ninghai	23 (3.32)	4 (1.49)	0.274 (0.088–0.854)*	0.186 (0.049–0.571)**
Yuyao	78 (11.27)	18 (6.72)	0.391 (0.207–0.738)**	0.372 (0.186–0.719)**
Cixi	89 (12.86)	31 (11.57)	0.696 (0.396–1.223)	0.642 (0.351–1.166)
Others	18 (2.60)	10 (3.73)	1.627 (0.601–4.407)	1.842 (0.633–5.549)
Region type				
Local city	524 (75.72)	208 (77.61)	1.000 (Reference)	
Other cities in this province	24 (3.47)	10 (3.73)	1.085 (0.473–2.489)	
Other provinces	144 (20.81)	50 (18.66)	0.808 (0.550–1.188)	
Transmission route				
Homosexual	302 (43.64)	126 (47.01)	1.000 (Reference)	
Heterosexual	387 (55.92)	141 (52.61)	0.801 (0.588–1.090)	
Others	3 (0.43)	1 (0.37)	0.698 (0.063–7.786)	–
Drug resistance				
Sensitive	644 (93.06)	256 (95.52)	1.000 (Reference)	1.000 (Reference)
Resistance	48 (6.94)	12 (4.48)	0.505 (0.258–0.989)*	0.451 (0.209–0.918)*
HIV-1 genotypes				
CRF01_AE	187 (27.02)	30 (11.19)	1.000 (Reference)	1.000 (Reference)
CRF07_BC	320 (46.24)	160 (59.70)	5.233 (3.344–8.190)***	6.016 (3.804–9.780)***
Others	185 (26.73)	78 (29.10)	3.815 (2.343–6.212)***	4.176 (2.488–7.155)***
Sample year				
2018	70 (10.12)	21 (7.84)	1.000 (Reference)	
2019	51 (7.37)	18 (6.72)	1.273 (0.590–2.746)	
2020	144 (20.81)	54 (20.15)	1.400 (0.759–2.583)	
2021	215 (31.07)	87 (32.46)	1.586 (0.889–2.830)	
2022	212 (30.64)	88 (32.84)	1.656 (0.927–2.956)	
Occupation				
Service sector	310 (44.80)	123 (45.90)	1.000 (Reference)	
Worker	122 (17.63)	49 (18.28)	1.020 (0.665–1.565)	
Former	156 (22.54)	60 (22.39)	0.950 (0.640–1.410)	
Others	104 (15.03)	36 (13.43)	0.805 (0.506–1.280)	
STD status				
Yes	149 (21.53)	53 (19.78)	1.000 (Reference)	
No	468 (67.63)	184 (68.66)	1.174 (0.800–1.722)	
Unknown	75 (10.84)	31 (11.57)	1.276 (0.722–2.254)	
Detection method				
Counseling and testing	143 (20.66)	58 (21.64)	1.000 (Reference)	
Physical examination	65 (9.39)	29 (10.82)	1.181 (0.653–2.134)	
STD clinic	108 (15.61)	37 (13.81)	0.764 (0.455–1.283)	
Focused survey	76 (10.98)	30 (11.19)	0.956 (0.541–1.687)	
General outpatient clinic	151 (21.82)	65 (24.25)	1.108 (0.697–1.761)	
Preoperative examination	149 (21.53)	49 (18.28)	0.718 (0.445–1.158)	
CD4 (cell/ul)				
<200	243 (35.13)	83 (31.01)	1.000 (Reference)	
≥200	449 (64.87)	185 (68.99)	1.347 (0.968–1.875)	

^a^≥5 sequence increase in the number of network sequences within a 1-year sequence was defined as fast-growing networks. *p < 0.05; **p < 0.01; ***p < 0.001.

### 3.5 Characterization of fast-growing clusters

**Figure 2:**
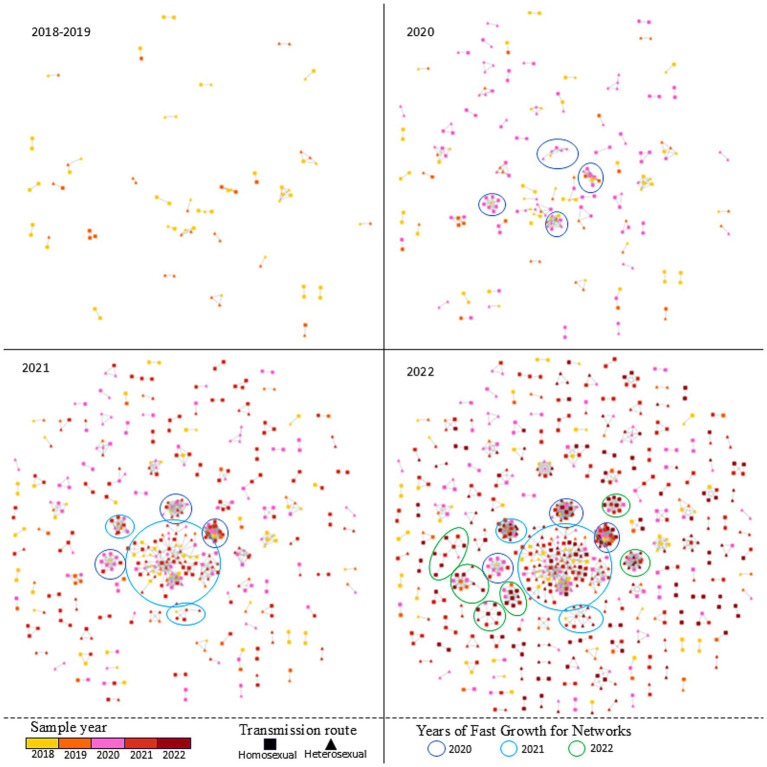
The temporal trends and Fast-growing clusters of HIV-1 transmission network in Ningbo (2018–2022).

To elucidate temporal trends, annual HIV-1 transmission networks were reconstructed for each study year (Figure 2). During 2020, four clusters emerged. By 2021, one cluster originating in 2020 had expanded significantly in size, while two smaller clusters formed independently. In 2022, five new small clusters appeared, and the previously expanded cluster continued to grow. Multivariate analysis identified underlying factors associated with network expansion dynamics. Residents from Ninghai (aOR = 0.186, 95% CI = 0.049–0.571, *p* = 0.006) and Yuyao (aOR = 0.372, 95% CI = 0.186–0.719, *p* = 0.004) exhibited reduced odds of clustering compared with Haishu residents. Drug-resistant individuals showed lower odds of involvement in rapid transmission networks (aOR = 0.451, 95% CI = 0.209–0.918, *p* = 0.034). Furthermore, infections with CRF07_BC (aOR = 6.016, 95% CI = 3.804–9.780, *p* < 0.001) and other subtypes (aOR = 4.176, 95% CI = 2.4889–7.155, *p* < 0.001) exhibited significantly stronger clustering propensities compared with CRF01_AE. Detailed information is listed in Supplementary Table 3.

## 4 Discussion

This study systematically investigated the molecular epidemiology, transmission dynamics, and risk factors of HIV-1 among 1,409 newly diagnosed, genotyped individuals in Ningbo City from 2018 to 2022. Molecular transmission network analysis revealed 168 distinct clusters involving 692 individuals, with 59.5% being dyadic (two-node) clusters and 9 clusters containing ≥10 nodes. Molecular transmission network analysis identified 9 large clusters and 12 fast-growing clusters. Age and HIV-1 subtype were associated with in the large clusters and fast-growing clusters, highlighting their critical roles in shaping local HIV transmission patterns. These findings provide critical insights into the structure and underlying factors associated with HIV transmission networks, which are essential for focused prevention and control strategies in Ningbo.

Previously, we analyzed molecular transmission networks in Ningbo from 2018 to 2021, revealing that the overall distribution of these networks remained relatively consistent over time. Specifically, CRF07_BC consistently tended to form larger, more densely connected clusters, whereas CRF01_AE networks primarily exhibited sparse, fragmented distributions. This aligns with nationwide trends in China, where CRF07_BC has shown increasing prevalence and CRF01_AE has declined in recent years (Li et al., 2016; Yin et al., 2019). Factors associated with cluster formation also showed consistency: CRF07_BC demonstrated a higher propensity to drive network formation during that period. Building on this foundation, the current study distinguishes itself by focusing specifically on the formation and influencing factors of large clusters and fast-growing clusters. While prior research emphasized the need for surveillance and comprehensive interventions focused on key clusters to mitigate potential HIV-1 transmission risks, it did not explicitly define or characterize what constituted these “key clusters.” In contrast, our work explicitly identifies large and fast-growing clusters as key areas for focused prevention and confirms that their formation remains consistently driven by HIV subtypes. Notably, CRF07_BC-associated clusters involved the most demographically heterogeneous populations, a pattern consistent with observations from Fujian, Anhui, and Jiangsu provinces (Wang et al., 2023; Zheng et al., 2021; Li et al., 2024). Similarly, analysis of fast-growing clusters reinforced the link between HIV-1 subtype and cluster dynamics: CRF07_BC showed a pronounced association with rapid network expansion. Structural analyses revealed that CRF07_BC harbors specific mutations/deletions in the p6Gag protein (e.g., PTAPPE insertion and/or PIDKELY deletion), which may attenuate virulence while enhancing transmissibility. Moreover, CRF07_BC variants with reduced net charge in the V3 loop exclusively utilize the CCR5 co-receptor and exhibit slower replication kinetics in primary target cells (Hu et al., 2022). These molecular features collectively suggest that CRF07_BC may possess superior fitness for initiating infections in key populations (Ge et al., 2021). In recent years, China has witnessed a bidirectional upward trend in HIV-1 infections, with notable increases among both adolescents and older adults. Our study revealed that older age (>55 years) was associated with a 2.38-fold higher likelihood of being included in MTNs compared to younger individuals—a pattern consistent with the national epidemiological trajectory (Ma et al., 2021; Cai et al., 2020). Similarly, a molecular transmission network analysis of HIV-1 in Huzhou, Zhejiang Province (2017–2022) highlighted that older adults (≥50 years) play a critical role in local network formation. This age-related disparity may be attributed to factors such as prolonged unprotected sexual encounters and a higher cumulative number of sexual partners among older individuals (Luo et al., 2019). However, our analysis revealed no age-related disparities in the formation of large clusters or fast-growing clusters. While prior research has established that distinct molecular transmission networks contribute disproportionately to HIV spread (Novitsky et al., 2023), we posit that the large clusters and fast-growing clusters prioritized for intervention in Ningbo should not be constrained by age. Given limited HIV prevention resources, prioritizing networks with greater future impact is imperative.

Beyond this, CRF57_BC—first identified in Yunnan Province (Li et al., 2012; Wei et al., 2014)—was absent from subtype surveys in Zhejiang during 2012–2016 (Ding et al., 2022) but was first reported in the province’s 2021 surveillance data (Fan et al., 2025). Notably, our study revealed that CRF57_BC had already formed large transmission networks by 2018–2022, a rapid dissemination pattern that warrants attention. Additionally, URFs constituted a large transmission network in Beilun District, one of China’s major seaports. Given the region’s unique geographical location and high population mobility—factors known to facilitate viral recombination between diverse subtypes—additional experimental validation is warranted to clarify these dynamics.

From a temporal perspective, our analysis showed that the number of sequences included in transmission networks in 2020 and 2021 was 1.57-fold and 1.77-fold higher, respectively, than that in 2018. However, no statistically significant difference was observed between 2022 and 2018. Visually, the MTNs expanded rapidly in 2020–2021 but plateaued in 2022—a divergent trend compared to other regions in Zhejiang Province and nationally, where networks have continued to grow unchecked. Ningbo features a well-developed medical information infrastructure and has allocated substantial financial and human resources to HIV prevention and control efforts. In 2020, Ningbo conducted a cost-effectiveness prediction for HIV interventions focused on MSM, projecting that scaling up intervention coverage by 3.0-fold (with a 2.4-fold increase in funding) from the 2020 baseline could reduce cumulative new HIV infections by 7.9% and AIDS-related deaths by 1.7% between 2021 and 2030 (Wang et al., 2022). Guided by this evidence, Ningbo has since intensified its prevention efforts through evidence-based strategies, including enhanced public education, optimized testing networks, improved treatment services, and technological innovation. Notable initiatives include the development of a medical-police collaborative big data system for efficient HIV source tracing, the “4 + 1” intensive management program for key HIV cases, and a risk prediction and early warning system for HIV transmission. These innovations were showcased at the 2024 “Fast-Track Cities” network symposium on ending the AIDS epidemic, where Ningbo shared its progress with domestic and international experts (Ningbo CDC, 2024). Collectively, these measures could have been instrumental in curbing the spread of HIV-1 in Ningbo.

In this study, we found no statistically significant association between drug resistance status and either sequences entered the transmission network or large clusters formed. However, sensitive sequences exhibited a significantly higher propensity to form fast-growing networks compared to their resistant sequences. While some prior studies have emphasized that factors such as HIV-1 subtype, antiretroviral treatment history, and transmission route critically influence drug resistance (Chen et al., 2023; Tan et al., 2023; Lan et al., 2021), evidence linking drug resistance itself to transmission network formation remains scarce. This gap may be attributed to China’s relatively low overall drug resistance rate, which has thus far prevented the emergence of large-scale, drug-resistant strain-dominated transmission networks. A study in Guangxi Province identified viral load (50–1,000 copies/mL) and immunological treatment failure as significant correlates of clustering (Chen et al., 2021). In contrast, U. S.-based research demonstrated that antiretroviral therapy (ART) focused on people living with HIV effectively curbed secondary HIV-1 transmission (Little et al., 2014). Similarly, a nationwide study in China reported that large clusters in Shenyang were controlled through ART interventions (Liu et al., 2020). However, in our study, neither CD4 count nor drug resistance factors yielded statistically significant associations with clustering. This observed discrepancy may be associated with Ningbo’s high ART coverage, which potentially saturates the preventive benefits derived from treatment-mediated viral suppression. Consistent with this hypothesis, a national HIV intervention prioritization analysis revealed that oral PrEP serves as an effective strategic intervention for MSM in regions with high ART coverage (e.g., Ningbo) under idealized scenarios (Zhang et al., 2024). MSM populations in cities like Beijing (Sun et al., 2023) and Nanjing (Chen et al., 2024) have shown high willingness to use PrEP; however, their actual adherence and usage rates remain extremely low (mostly <5%) (Du et al., 2025; Peng et al., 2022). Three key scenarios may contribute to drug resistance during PrEP use: initiating PrEP without prior HIV diagnosis or awareness of infection status, inconsistent adherence to PrEP medications, and resuming high-risk behaviors shortly after discontinuing PrEP while residual prophylactic drugs remain in the body. All of these scenarios can lead to the acquisition of drug-resistant HIV. Research has further shown that drug resistance is particularly prevalent among individuals who start PrEP during acute HIV infection without a confirmed diagnosis; however, widespread PrEP implementation with sustained high adherence does not significantly increase the transmission of resistant strains (Johnson et al., 2021). To date, Ningbo has not implemented focused PrEP policies, and no local studies have investigated PrEP adoption. Only information on post-exposure prophylaxis (PEP) clinics is publicly available on the Ningbo Center for Disease Control and Prevention (CDC) website. We therefore hypothesize that PrEP usage in Ningbo is minimal, and its potential confounding effect on our drug resistance findings is likely negligible.

This study has several limitations that should be acknowledged. First, the analysis was restricted to newly diagnosed people living with HIV with successful genotype data, potentially introducing selection bias by excluding cases with poor-quality or unsequenced samples. This may constrain the generalizability of findings to the broader community of people living with HIV in Ningbo, particularly among individuals with undiagnosed or unsequenced infections. Second, the study focused on a 5-year period (2018–2022), which may not capture long-term trends or the impact of very recent intervention adjustments (e.g., post-2022 policy changes). Third, while molecular transmission networks were constructed using genetic sequence data, the analysis did not integrate detailed behavioral or socioeconomic factors (e.g., sexual partner dynamics, socioeconomic status) that may influence transmission patterns, potentially overlooking factors associated with cluster formation. Fourth, the evaluation of intervention effectiveness relied on observational data, which may be confounded by concurrent public health initiatives or regional disparities in service access, making causal inferences challenging. Finally, the findings are specific to Ningbo’s unique epidemiological and geographic context (e.g., port-related population mobility), limiting direct extrapolation to other cities or regions with distinct demographic, behavioral, or structural features. Future studies should address these gaps by expanding sample diversity, incorporating longitudinal behavioral data, and exploring external validity through multi-region comparisons.

## 5 Conclusion

This study systematically characterized the molecular transmission dynamics of HIV-1 in Ningbo City from 2018 to 2022. Key findings revealed that the CRF07_BC subtype exhibited a significant propensity to form large transmission clusters and rapidly expanding transmission clusters; adults aged ≥55 years played a critical role in the formation of local transmission networks. Continuous monitoring of HIV-1 molecular subtypes may inform feasible and focused prevention strategies to curb HIV transmission.

## Data Availability

The data presented in the study are deposited in GenBank, accession number PX482927 to PX484313.

## Footnotes

^1^Los Alamos National Laboratory, LANL; www.hiv.lanl.gov.

^2^https://www.hiv.lanl.gov/content/sequence/elimdupesv2/elimdupes.html.

^3^https://hivdb.stanford.edu.
