# Supplementary material for: Molecular transmission network analysis of newly diagnosed HIV-1 infections in Ningbo from 2018–2022
Source: Front Microbiol. 2025 Nov 19;16:1701408. doi: 10.3389/fmicb.2025.1701408 (PMC12672912; doi:10.3389/fmicb.2025.1701408)
Supplement: Supplementary file 1 [file Data_Sheet_1.docx]

​​Supplementary Files

Supplementary Methods​

Recognizing that this threshold may not universally apply due to variations in viral subtype diversity and epidemiological dynamics^1,2^, we adopted a data-driven approach. Using HyPhy 2.2.4 software, we calculated pairwise TN93 genetic distances and systematically evaluated cluster formation across a range of thresholds. By identifying the genetic distance that maximized the number of discernible transmission clusters while balancing sensitivity and specificity, we determined that thresholds of 0.012 and 0.013 yielded the highest cluster counts. To ensure methodological continuity and comparability with our prior study^3^ (which utilized a 0.013 threshold), we retained this value for the current investigation.


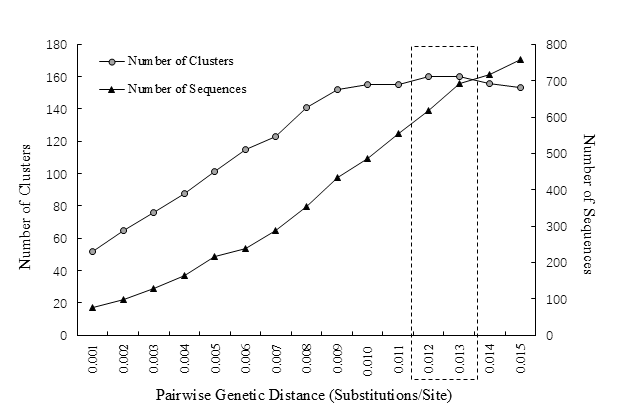


**Figure S1**. Clustering under different thresholds.

1. Hassan AS, Pybus OG, Sanders EJ, Albert J, Esbjörnsson J. Defining HIV-1 transmission clusters based on sequence data. *AIDS*. 2017;31(9):1211-1222. doi:10.1097/QAD.0000000000001470
2. Cheng P, He BC, Liu JF, et al. Using the Molecular Transmission Networks to Analyze the Epidemic Characteristics of HIV-1 *CRF08_BC* in Kunming, Yunnan. *AIDS Res Hum Retroviruses*. 2024;40(5):353-362. doi:10.1089/AID.2023.0060
3. Hong H, Tang C, Liu Y, Jiang H, Fang T, Xu G. HIV-1 drug resistance and genetic transmission network among newly diagnosed people living with HIV/AIDS in Ningbo, China between 2018 and 2021. *Virol J*. 2023;20(1):233. Published 2023 Oct 13. doi:10.1186/s12985-023-02193-x

**Table S1**. The list of reference sequences.

| Subtypes | Country | Sampling year | Accession |
| --- | --- | --- | --- |
| CRF01_AE | CN | 2005 | GU564221 |
| CRF01_AE | CN | 2005 | MW079295 |
| CRF01_AE | CN | 2007 | FM251979 |
| CRF01_AE | CN | 2009 | JX960603 |
| CRF01_AE | CN | 2009 | JX112870 |
| CRF01_AE | CN | 2010 | JX112797 |
| CRF01_AE | CN | 2010 | JX112849 |
| CRF01_AE | CN | 2013 | KM258698 |
| CRF01_AE | CN | 2013 | MW956272 |
| CRF01_AE | CN | 2014 | MN425263 |
| CRF01_AE | CN | 2016 | MW950403 |
| CRF07_BC | CN | 2006 | JX392378 |
| CRF07_BC | CN | 2007 | KF250385 |
| CRF07_BC | CN | 2008 | KU050503 |
| CRF07_BC | CN | 2009 | KJ820233 |
| CRF07_BC | CN | 2009 | KT378991 |
| CRF07_BC | CN | 2009 | KJ820384 |
| CRF07_BC | CN | 2012 | KT893578 |
| CRF07_BC | CN | 2012 | MN426180 |
| CRF07_BC | CN | 2012 | KP418626 |
| CRF07_BC | CN | 2014 | KX306117 |
| CRF08_BC | CN | 2007 | KF835497 |
| CRF08_BC | CN | 2012 | MN908847 |
| CRF08_BC | CN | 2013 | MW956245 |
| CRF55_01B | CN | 2010 | MN427046 |
| CRF55_01B | CN | 2014 | MW956857 |
| CRF57_BC | CN | 2009 | JX679207 |
| CRF59_01B | CN | 2019 | MW573933 |
| CRF64_BC | CN | 2010 | KC870040 |
| CRF67_01B | CN | 2011 | KC183779 |
| CRF67_01B | CN | 2011 | KC183780 |
| CRF68_01B | CN | 2010 | KF758551 |
| CRF68_01B | CN | 2011 | KC183783 |
| CRF85_BC | CN | 2014 | KU992937 |
| B | CN | 2001 | AB750367 |
| B | CN | 2018 | MN633570 |
| C | IN | 1993 | AB023804 |
| URF_0107 | CN | 2019 | MZ359115 |
| URF_0708 | CN | 2000 | AP005207 |
| URF_BC | CN | 2000 | AB078691 |
| URF_01BC | CN | 2013 | KU378046 |
| URF_01BC | CN | 2018 | MZ159978 |
| URF_01BC | TH | 2011 | MT121818 |
| O_Out Group | US | 1997 | AY169805 |

**Table S2**. Characteristics of study participants between different HIV-1 genotypes

| Characteristic | All  N = 1409(%) | | CRF01_AE  N = 468(%) | CRF07_BC  N = 586(%) | Others  N = 355(%) | *P* |
| --- | --- | --- | --- | --- | --- | --- |
| Gender |  |  | |  |  | <0.001 |
| Male | 1,198 (85.02) | 398 (85.04) | | 518 (88.40) | 282 (79.44) |  |
| Female | 211 (14.98) | 70 (14.96) | | 68 (11.60) | 73 (20.56) |  |
| Age (group) |  |  | |  |  | 0.239 |
| ≤25 | 198 (14.05) | 65 (13.89) | | 95 (16.21) | 38 (10.70) |  |
| 25-35 | 365 (25.90) | 123 (26.28) | | 153 (26.11) | 89 (25.07) |  |
| 35-45 | 245 (17.39) | 79 (16.88) | | 104 (17.75) | 62 (17.46) |  |
| 45-55 | 285 (20.23) | 94 (20.09) | | 120 (20.48) | 71 (20.00) |  |
| >55 | 316 (22.43) | 107 (22.86) | | 114 (19.45) | 95 (26.76) |  |
| Marital status |  |  | |  |  | 0.569 |
| Single | 527 (37.40) | 178 (38.03) | | 228 (38.91) | 121 (34.08) |  |
| Married | 602 (42.73) | 198 (42.31) | | 240 (40.96) | 164 (46.20) |  |
| Divorce or death | 280 (19.87) | 92 (19.66) | | 118 (20.14) | 70 (19.72) |  |
| Education level |  |  | |  |  | 0.382 |
| Primary school or below | 363 (25.76) | 116 (24.79) | | 139 (23.72) | 108 (30.42) |  |
| Junior high school | 477 (33.85) | 158 (33.76) | | 201 (34.30) | 118 (33.24) |  |
| Senior high school or secondary vocational school | 245 (17.39) | 85 (18.16) | | 103 (17.58) | 57 (16.06) |  |
| Associate degree or above | 324 (23.00) | 109 (23.29) | | 143 (24.40) | 72 (20.28) |  |
| ​​Ethnic group |  |  | |  |  | 0.166 |
| Han | 1,348 (95.67) | 453 (96.79) | | 561 (95.73) | 334 (94.08) |  |
| Others | 61 (4.33) | 15 (3.21) | | 25 (4.27) | 21 (5.92) |  |
| Affiliated Region |  |  | |  |  | <0.001 |
| Haishu | 267 (18.95) | 103 (22.01) | | 107 (18.26) | 57 (16.06) |  |
| Jiangbei | 53 (3.76) | 16 (3.42) | | 25 (4.27) | 12 (3.38) |  |
| Beilun | 129 (9.16) | 33 (7.05) | | 46 (7.85) | 50 (14.08) |  |
| Zhenhai | 55 (3.90) | 16 (3.42) | | 25 (4.27) | 14 (3.94) |  |
| Yinzhou | 290 (20.58) | 108 (23.08) | | 128 (21.84) | 54 (15.21) |  |
| Fenghua | 73 (5.18) | 24 (5.13) | | 36 (6.14) | 13 (3.66) |  |
| Xiangshan | 86 (6.10) | 19 (4.06) | | 27 (4.61) | 40 (11.27) |  |
| Ninghai | 51 (3.62) | 14 (2.99) | | 25 (4.27) | 12 (3.38) |  |
| Yuyao | 166 (11.78) | 61 (13.03) | | 66 (11.26) | 39 (10.99) |  |
| Cixi | 191 (13.56) | 56 (11.97) | | 84 (14.33) | 51 (14.37) |  |
| Others | 48 (3.41) | 18 (3.85) | | 17 (2.90) | 13 (3.66) |  |
| Region Type |  |  | |  |  | 0.580 |
| Local city | 1,018 (72.25) | 329 (70.30) | | 422 (72.01) | 267 (75.21) |  |
| Other cities in this province | 48 (3.41) | 18 (3.85) | | 21 (3.58) | 9 (2.54) |  |
| Other provinces | 343 (24.34) | 121 (25.85) | | 143 (24.40) | 79 (22.25) |  |
| Transmission route |  |  | |  |  | - |
| Homosexual | 646 (45.85) | 200 (42.74) | | 300 (51.19) | 146 (41.13) |  |
| Heterosexual | 757 (53.73) | 266 (56.84) | | 283 (48.29) | 208 (58.59) |  |
| Others | 6 (0.43) | 2 (0.43) | | 3 (0.51) | 1 (0.28) |  |
| Drug resistance |  |  | |  |  | 0.015 |
| Sensitive | 1,298 (92.12) | 421 (89.96) | | 554 (94.54) | 323 (90.99) |  |
| Resistance | 111 (7.88) | 47 (10.04) | | 32 (5.46) | 32 (9.01) |  |
| Sample year |  |  | |  |  | 0.439 |
| 2018 | 165 (11.71) | 49 (10.47) | | 78 (13.31) | 38 (10.70) |  |
| 2019 | 112 (7.95) | 45 (9.62) | | 45 (7.68) | 22 (6.20) |  |
| 2020 | 282 (20.01) | 102 (21.79) | | 109 (18.60) | 71 (20.00) |  |
| 2021 | 393 (27.89) | 128 (27.35) | | 166 (28.33) | 99 (27.89) |  |
| 2022 | 457 (32.43) | 144 (30.77) | | 188 (32.08) | 125 (35.21) |  |
| CD4 (cells/μL) |  |  | |  |  | <0.001 |
| <200 | 512 (37.81) | 204 (45.43) | | 185 (32.74) | 123 (36.18) |  |
| ≥200 | 842 (62.19) | 245 (54.57) | | 380 (67.26) | 217 (63.82) |  |
| Occupation |  |  | |  |  | 0.004 |
| Service sector | 617 (43.79) | 193 (41.24) | | 273 (46.59) | 151 (42.54) |  |
| Worker | 271 (19.23) | 77 (16.45) | | 120 (20.48) | 74 (20.85) |  |
| Former | 313 (22.21) | 114 (24.36) | | 107 (18.26) | 92 (25.92) |  |
| Others | 208 (14.76) | 84 (17.95) | | 86 (14.68) | 38 (10.70) |  |
| Detection Method |  |  | |  |  | 0.049 |
| Counseling and testing | 286 (20.30) | 82 (17.52) | | 126 (21.50) | 78 (21.97) |  |
| Physical examination | 141 (10.01) | 55 (11.75) | | 60 (10.24) | 26 (7.32) |  |
| STD clinic | 226 (16.04) | 79 (16.88) | | 85 (14.51) | 62 (17.46) |  |
| Targeted survey | 151 (10.72) | 39 (8.33) | | 78 (13.31) | 34 (9.58) |  |
| General outpatient clinic | 310 (22.00) | 102 (21.79) | | 129 (22.01) | 79 (22.25) |  |
| Preoperative examination | 295 (20.94) | 111 (23.72) | | 108 (18.43) | 76 (21.41) |  |
| STD Status |  |  | |  |  | 0.892 |
| Yes | 294 (20.87) | 101 (21.58) | | 116 (19.80) | 77 (21.69) |  |
| No | 975 (69.20) | 322 (68.80) | | 413 (70.48) | 240 (67.61) |  |
| Unknown | 140 (9.94) | 45 (9.62) | | 57 (9.73) | 38 (10.70) |  |


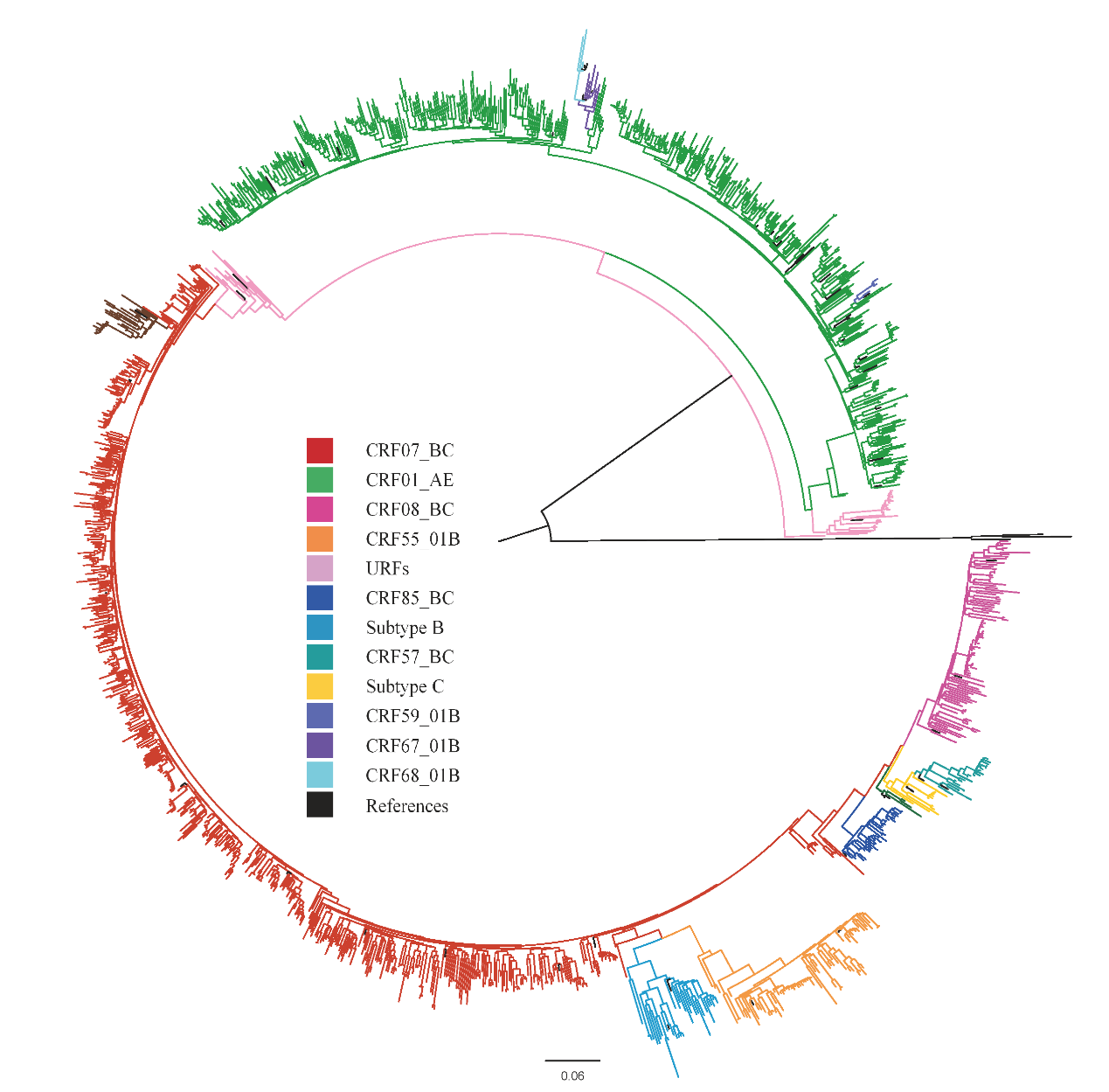


**Figure S2**: Phylogenetic tree analysis of nucleotide sequences from people living with HIV in Ningbo.


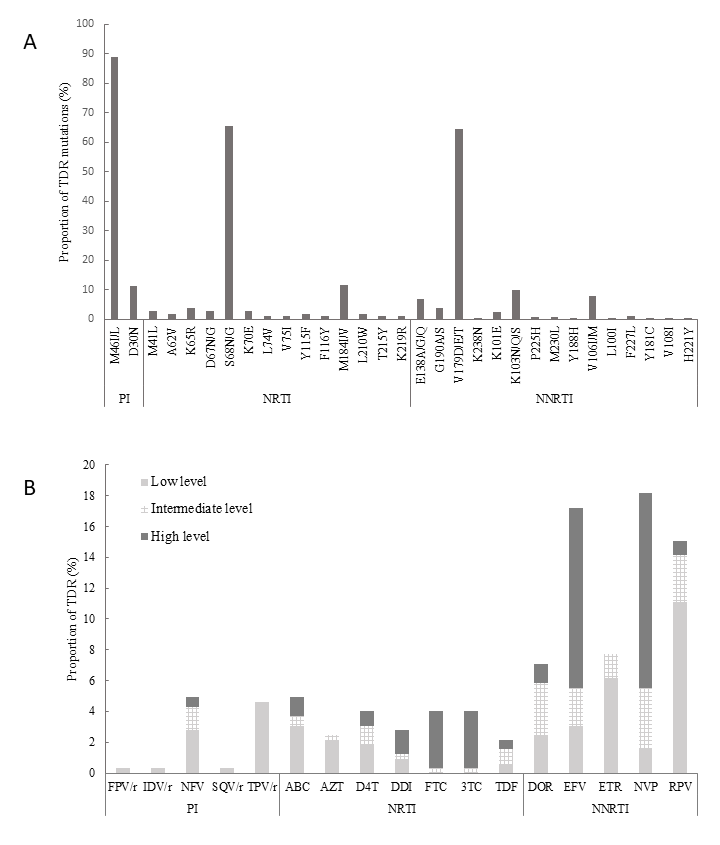


**Figure S3**.​​ (A) Proportions of HIV-1 transmitted drug resistance (TDR) mutations targeting PIs, NRTIs, and NNRTIs. (B) Frequencies of HIV-1 TDR-associated mutations across different antiretroviral therapy (ART) drug classes in 1,006 people living with HIV. ​​Abbreviations:​​ PI, Protease inhibitor; NRTI, Nucleoside reverse transcriptase inhibitor; NNRTI, Non-nucleoside reverse transcriptase inhibitor; NFV, Nelfinavir; ABC, Abacavir; AZT, Zidovudine; D4T, Stavudine; DDI, Didanosine; FTC, Emtricitabine; 3TC, Lamivudine; TDF, Tenofovir; DOR, Doravirine; EFV, Efavirenz; ETR, Etravirine; RPV, Rilpivirine.


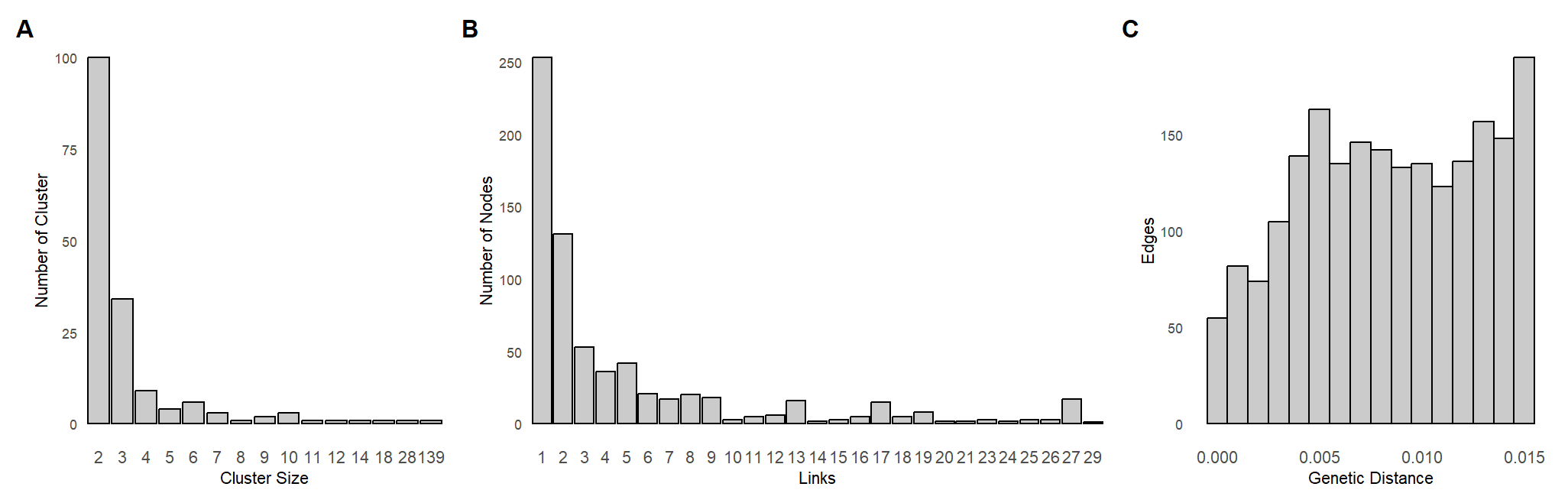


**Figure S4**. The characteristics of the molecular transmission networks. (A) Distribution of molecular transmission clusters by cluster size; (B) Distribution of nodes in clusters by links; (C) Distribution of edges by difference genetic distances.


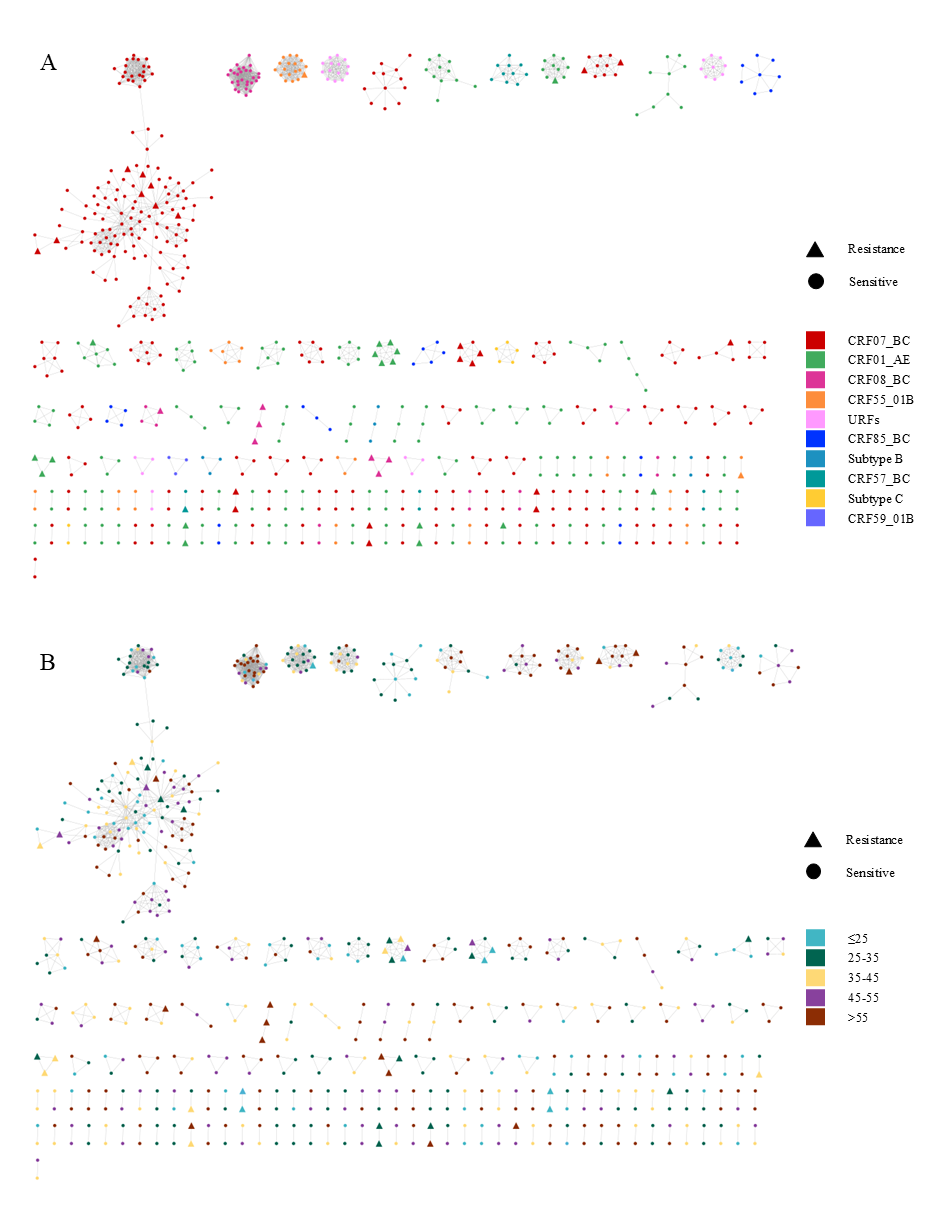


**Figure S5**. Transmission networks and drug resistance profiles of people living with HIV in Ningbo (2018–2022).​​ Clusters are ordered by size within each panel. Node shapes denote distinct transmission routes, and node colors represent participant characteristics, including HIV-1 genotypes and age groups.

**Table S3**. Factors associated with study participants within genetic transmission networks in Ningbo

| Characteristic | Participants  N = 1409 (%) | Clustered  N = 692(%) | Univariate analysis | | Multivariate analysis |
| --- | --- | --- | --- | --- | --- |
|  |  |  | OR (95%CI) | OR (95%CI) | |
| Gender |  |  |  |  | |
| Male | 1,198 (85.02) | 580 (83.82) | 1.000 (Reference) |  | |
| Female | 211 (14.98) | 112 (16.18) | 1.136(0.859 - 1.503) |  | |
| Age (group) |  |  |  |  | |
| <=25 | 198 (14.05) | 89 (12.86) | 1.000 (Reference) | 1.000 (Reference) | |
| 25-35 | 365 (25.90) | 175 (25.29) | 1.022(0.729 - 1.433) | 1.248（0.868 - 1.797) | |
| 35-45 | 245 (17.39) | 119 (17.20) | 1.033(0.718 - 1.487) | 1.288（0.870 – 1.909) | |
| 45-55 | 285 (20.23) | 112 (16.18) | 0.801(0.558 - 1.149) | 0.863（0.585 – 1.273) | |
| >55 | 316 (22.43) | 197 (28.47) | 1.923(1.354 - 2.730)*** | 2.379（1.623 – 3.502)*** | |
| Marital status |  |  |  |  | |
| Single | 527 (37.40) | 247 (35.69) | 1.000 (Reference) |  | |
| Married | 602 (42.73) | 317 (45.81) | 1.287(1.026 - 1.614)* |  | |
| Divorce or death | 280 (19.87) | 128 (18.50) | 0.965(0.728 - 1.279) |  | |
| Education level |  |  |  |  | |
| Primary school or below | 363 (25.76) | 203 (29.34) | 1.000 (Reference) |  | |
| Junior high school | 477 (33.85) | 215 (31.07) | 0.655(0.502 - 0.854)** |  | |
| Senior high school or secondary vocational school | 245 (17.39) | 122 (17.63) | 0.775(0.566 - 1.060) |  | |
| Associate degree or above | 324 (23.00) | 152 (21.97) | 0.668(0.500 - 0.893)** |  | |
| ​​Ethnic group |  |  |  |  | |
| Han | 1,348 (95.67) | 671 (96.97) | 1.000 (Reference) | 1.000 (Reference) | |
| Others | 61 (4.33) | 21 (3.03) | 0.531(0.313 - 0.898)* | 0.494（0.270 - 0.873)* | |
| Affiliated Region |  |  |  |  | |
| Haishu | 267 (18.95) | 122 (17.63) | 1.000 (Reference) |  | |
| Jiangbei | 53 (3.76) | 22 (3.18) | 0.923(0.513 - 1.662) |  | |
| Beilun | 129 (9.16) | 62 (8.96) | 1.227(0.811 - 1.855) |  | |
| Zhenhai | 55 (3.90) | 30 (4.34) | 1.433(0.818 - 2.511) |  | |
| Yinzhou | 290 (20.58) | 153 (22.11) | 1.308(0.949 - 1.803) |  | |
| Fenghua | 73 (5.18) | 38 (5.49) | 1.423(0.855 - 2.367) |  | |
| Xiangshan | 86 (6.10) | 57 (8.24) | 2.256(1.395 - 3.649)*** |  | |
| Ninghai | 51 (3.62) | 23 (3.32) | 0.937(0.556 - 1.671) |  | |
| Yuyao | 166 (11.78) | 78 (11.27) | 1.091(0.748 - 1.591) |  | |
| Cixi | 191 (13.56) | 89 (12.86) | 0.986(0.690 - 1.411) |  | |
| Others | 48 (3.41) | 18 (2.60) | 0.756(0.407 - 1.404) |  | |
| Region Type |  |  |  |  | |
| Local city | 1,018 (72.25) | 524 (75.72) | 1.000 (Reference) |  | |
| Other cities in this province | 48 (3.41) | 24 (3.47) | 1.061(0.598 - 1.880) |  | |
| Other provinces | 343 (24.34) | 144 (20.81) | 0.717(0.563 - 0.912)** |  | |
| Transmission route |  |  |  |  | |
| Homosexual | 646 (45.85) | 302 (43.64) | 1.000 (Reference) |  | |
| Heterosexual | 757 (53.73) | 387 (55.92) | 1.174(0.958 - 1.439) |  | |
| Others | 6 (0.43) | 3 (0.43) | 0.976(0.217 - 4.393) | - | |
| Drug resistance |  |  |  |  | |
| Sensitive | 1,298 (92.12) | 644 (93.06) | 1.000 (Reference) |  | |
| Resistance | 111 (7.88) | 48 (6.94) | 0.774(0.523 - 1.144) |  | |
| HIV-1 genotypes |  |  |  |  | |
| CRF01_AE | 468(30.83) | 187 (27.02) | 1.000 (Reference) | 1.000 (Reference) | |
| CRF07_BC | 586(38.60) | 320 (46.24) | 1.808(1.413 - 2.313)*** | 1.806（1.395 - 2.342)*** | |
| Others | 355(23.39) | 185 (26.73) | 1.635(1.238 - 2.160)*** | 1.577（1.177 - 2.115)** | |
| Sample year |  |  |  |  | |
| 2018 | 165 (11.71) | 70 (10.12) | 1.000 (Reference) | 1.000 (Reference) | |
| 2019 | 112 (7.95) | 51 (7.37) | 1.397(0.869 - 2.248) | 1.087（0.658 - 1.794) | |
| 2020 | 282 (20.01) | 144 (20.81) | 1.660(1.140 - 2.416)** | 1.571（1.055 - 2.349)* | |
| 2021 | 393 (27.89) | 215 (31.07) | 1.843(1.294 - 2.625)*** | 1.765（1.208 - 2.589)** | |
| 2022 | 457 (32.43) | 212 (30.64) | 1.150(0.816 - 1.623) | 1.242（0.856 - 1.809) | |
| Occupation |  |  |  |  | |
| Service sector | 617 (43.79) | 310 (44.80) | 1.000 (Reference) |  | |
| Worker | 271 (19.23) | 122 (17.63) | 0.840(0.635 - 1.110) |  | |
| Former | 313 (22.21) | 156 (22.54) | 0.937(0.722 - 1.217) |  | |
| Others | 208 (14.76) | 104 (15.03) | 0.984(0.726 - 1.333) |  | |
| STD Status |  |  |  |  | |
| Yes | 294 (20.87) | 149 (21.53) | 1.000 (Reference) |  | |
| No | 975 (69.20) | 468 (67.63) | 0.861(0.668 - 1.109) |  | |
| Unknown | 140 (9.94) | 75 (10.84) | 1.117(0.755 - 1.652) |  | |
| Detection Method |  |  |  |  | |
| Counseling and testing | 286 (20.30) | 143 (20.66) | 1.000 (Reference) |  | |
| Physical examination | 141 (10.01) | 65 (9.39) | 0.920(0.621 - 1.364) |  | |
| STD clinic | 226 (16.04) | 108 (15.61) | 0.947(0.676 - 1.327) |  | |
| Targeted survey | 151 (10.72) | 76 (10.98) | 1.026(0.702 - 1.501) |  | |
| General outpatient clinic | 310 (22.00) | 151 (21.82) | 0.986(0.722 - 1.345) |  | |
| Preoperative examination | 295 (20.94) | 149 (21.53) | 1.006(0.736 - 1.375) |  | |
| CD4 (cell/ul) |  |  |  |  | |
| <200 | 512 (37.81) | 243 (35.13) | 1.000 (Reference) | 1.000 (Reference) | |
| ≥200 | 842 (62.19) | 449 (64.87) | 1.186(0.958 - 1.469) | 1.295（1.023 – 1.641)** | |

* *P*<0.05

** *P*<0.01

*** *P*<0.001
